# Seasonal activity, vector relationships and genetic analysis of mosquito-borne Stratford virus

**DOI:** 10.1371/journal.pone.0173105

**Published:** 2017-03-02

**Authors:** Cheryl S. Toi, Cameron E. Webb, John Haniotis, John Clancy, Stephen L. Doggett

**Affiliations:** 1 Department of Medical Entomology, Centre for Infectious Diseases and Microbiology Laboratory Services, NSW Health Pathology, Westmead Hospital, Westmead, New South Wales, Australia; 2 Marie Bashir Institute for Infectious Diseases and Biosecurity, University of Sydney, New South Wales, Australia; University of California Davis, UNITED STATES

## Abstract

There are many gaps to be filled in our understanding of mosquito-borne viruses, their relationships with vectors and reservoir hosts, and the environmental drivers of seasonal activity. Stratford virus (STRV) belongs to the genus *Flavivirus* and has been isolated from mosquitoes and infected humans in Australia but little is known of its vector and reservoir host associations. A total of 43 isolates of STRV from mosquitoes collected in New South Wales between 1995 and 2013 was examined to determine the genetic diversity between virus isolates and their relationship with mosquito species. The virus was isolated from six mosquito species; *Aedes aculeatus*, *Aedes alternans*, *Aedes notoscriptus*, *Aedes procax*, *Aedes vigilax*, and *Anopheles annulipes*. While there were distinct differences in temporal and spatial activity of STRV, with peaks of activity in 2006, 2010 and 2013, a sequence homology of 95.9%–98.4% was found between isolates and the 1961 STRV prototype with 96.2%–100% identified among isolates. Temporal differences but no apparent nucleotide divergence by mosquito species or geographic location was evident. The result suggests the virus is geographically widespread in NSW (albeit only from coastal regions) and increased local STRV activity is likely to be driven by reservoir host factors and local environmental conditions influencing vector abundance. While STRV may not currently be associated with major outbreaks of human disease, with the potential for urbanisation and climate change to increase mosquito-borne disease risks, and the possibility of genomic changes which could produce pathogenic strains, understanding the drivers of STRV activity may assist the development of strategic response to public health risks posed by zoonotic flaviviruses in Australia.

## Introduction

The strategic response of local authorities to the risk of mosquito-borne disease requires an understanding of the environmental, entomological, and ecological drivers of virus activity [1]. While there are over 70 arboviruses known to occur in the Australian region [2, 3], relatively few pose serious human health risks [4]. The alphaviruses, Ross River virus (RRV) and Barmah Forest virus (BFV) are the most common mosquito-borne pathogens causing human disease with around 6,000 cases officially notified across Australia each year [4]. However, endemic mosquito-borne flaviviruses including Murray Valley encephalitis virus (MVEV) and the Kunjin strain of West Nile virus (WNV_KUN_) have the potential to cause serious human and veterinary health risks [5, 6]. Amid possible increases in future mosquito-borne disease risk associated with a changing climate, anthropogenic effects that result in increased vector/human contact, potential genomic changes that can increase the virulence of viruses, and the likely introduction of exotic vectors [7, 8], it is important to fill the gaps in our understanding of relationships between pathogens, vectors, reservoir hosts and environmental conditions to determine the key drivers of public health risks.

Stratford virus (STRV) belongs to the Kokobera subgroup of the genus *Flavivirus* and the family Flaviviridae [9]. It was first isolated in 1961 from *Aedes vigilax* Skuse collected in Cairns, Far North Queensland [10] and is endemic in Australia. The clinical role and importance of STRV to human health has not been fully defined, although serological evidence of past human infections have been identified during a serosurvey conducted in south-eastern Australia in 1985/86 that reported serum antibodies to STRV [11, 12]. At the time of this serosurvey, no isolations of STRV from mosquitoes were reported because no mosquito or mosquito-borne pathogen surveillance program existed. Subsequently, STRV has been isolated from the *Aedes* species of mosquitoes from a number of locations in south east Australia [2, 13]. There is no information available on reservoir hosts [2], other than the *Aedes* species display a wide host-feeding preference [13, 14].

*Aedes vigilax* is one of the most important pest and vector mosquitoes in Australia and commonly associated with tidally influenced estuarine wetlands in all but the most southern coastal regions of Australia [4]. Populations of this mosquito can be extraordinarily abundant and, notwithstanding the nuisance-biting impacts on coastal communities, there is strong evidence that this mosquito plays an important role in the transmission of arboviruses within these regions. *Aedes procax*, however, is closely associated with freshwater to brackish water habitats, particularly coastal swamp forests [15] and can be locally abundant. It has also been shown to transmit a range of arboviruses in the laboratory [16, 17]. *Aedes notoscriptus* is closely associated with natural and artificial water-holding containers and is widespread across urban areas of Australia [18]. It is a nuisance-biting pest and arboviruses have been isolated from field collected specimens [13]. This mosquito is shown to be an efficient vector of RRV and BFV in laboratory studies [19–21] but does not disperse widely from larval habitats [22].

Study on the geographic variation in pathogen genotype provide insights into the reservoir hosts of arboviruses and subsequently, may assist in the development of surveillance programs and epidemic early warnings systems. Phylogenetic studies of two endemic flaviviruses, MVEV and WNV_KUN_, have already shown relative homogeneity within the viral type and a close association with mobile reservoir hosts such as birds. However, the endemic alphavirus RRV has shown distinct geographic variation with greater genetic variability. Subsequently, two topotypes have been identified from macropods which are relatively sedentary reservoir hosts [23]. The role of macropods as important reservoir hosts has been implicated through serological surveys [24]. This trend is not consistent across other Australian alphaviruses studied to date with the genetic variability of Sindbis virus (SINV) found to vary temporally, rather than geographically across 40 isolates studied; it was proposed that this temporal clustering suggests migratory avian hosts and that they may rapidly disperse the virus from enzootic focal points [25]. Similarly, BFV displays a high degree of sequence homology suggesting little geographic or temporal divergence, consistent with avian reservoir hosts [26]. However, while genetic analysis of mosquito-borne viruses may suggest mobile reservoirs hosts, it is important to note that as well as avian hosts, flying mammals (e.g. bats) may also play a role in geographic dispersal of pathogens [6, 27].

Hence, the aim of this study was to investigate the genetic diversity between isolates of STRV derived from field collected mosquito specimens. This will determine if there are any temporal, geographic, or mosquito-specific relationships that may suggest entomological or wildlife drivers of arbovirus activity.

## Materials and methods

### Mosquito collection

All isolates of STRV were cultured from field collected mosquito specimens. These collections were undertaken between 1995 and 2013 as part of the NSW Arbovirus Surveillance and Mosquito Monitoring program that runs on a weekly basis between November and April at various locations throughout NSW [13]. Adult mosquitoes are collected using dry-ice baited encephalitis virus surveillance (EVS) traps [28] with specimens identified to species using the taxonomic keys of Russell (1993) [29].

### Virus isolation and identification

Viruses were isolated via cell culture and identified using a Fixed-Cell ELISA [30]. Briefly, pools of up to 25 mosquitoes of the same species were placed into tubes containing 2 mL of RPMI 1640 (Roswell Park Memorial Institute medium), antibiotics, foetal calf serum and glass beads and shaken for 20 minutes at 4°C until ground. The samples were clarified at 4000 rpm for 20 minutes at 4°C and the supernatants inoculated onto C6/36 insect cells and incubated for 3–4 days for the initial replication of virus. An inoculum was transferred onto C6/36 and baby hamster kidney (BHK) cells and incubated for a further 3–4 days. The purpose was to increase the infectious titres of slower growing flaviviruses in C6/36 cells and to observe cytopathic effect (CPE) in mammalian cells. Culture supernatant from infected mammalian cells showing virus CPE were processed for sequencing. Positive isolates were identified by FC ELISA using a panel of flavivirus-specific MAbs, 1E3 and 2E5 that differentiates between Stratford virus from KOKV and other Kokobera-like viruses [31].

### RT-PCR and DNA sequencing

Viral RNA was extracted from 200 μL of cell culture supernatant using the EZ1^®^ Virus Mini Kit v2.0 on the BioRobot^®^ (Qiagen, Limburg, Netherlands) in accordance with the manufacturers’ instruction. A two-step RT-PCR was performed using Tetro Reverse Transcriptase (Bioline, Sydney, Australia) and random hexamer primers (Roche, Mannheim, Germany) for cDNA synthesis. The following cycling conditions were applied: 22°C for 5 min, 50°C for 35 min and 70°C for 7 min on the Veriti^®^ Thermal Cycler (Applied Biosystems, California, USA).

Universal primers spanning an 802 bp region encoding part of the methyltransferase and the start of the region encoding RNA-dependent-RNA polymerase in the flavivirus non- structural protein 5 (NS5) gene sequence were selected for PCR [32]. These comprised of consensus degenerate bases; Flav100F (5’-AAYTCNACNCANGARATGTAYT-3’) and reverse primer Flav200R (5’-CCNARCCACATRWACCA-3’). The primer sequences and their positions (8276–9078) are relative to the Yellow fever virus genome, GenBank reference NC_002031.

The 802 bp fragment was amplified by real-time PCR on the Rotogene 6000 (Qiagen, Victoria, Australia) in a total volume of 20 μL containing 1.8 μL of cDNA template, 300 nM of each primer, 1 × of 20-fold EvaGreen (Biotium, CA, USA), 1 × My Taq reaction buffer, a proprietary formulation containing dNTPs, MgCl_2_ and enhancers, 1U of MyTaq^™^ HS DNA polymerase (Bioline, Sydney, Australia) and RNase- and DNAse-free water (Sigma, St. Louis, USA). The thermal cycling commenced with enzyme activation (95°C for 1 min), followed by 40 cycles of denaturation (95°C for 20 s), annealing (51°C for 30 s), extension (72°C for 40 s), and a final extension at 72°C for 2 min. A pre-hold cycle was set at 50°C for 30 s followed by a melt cycle with ramping temperatures between 75°C and 95°C to serve as a check on purity of the amplified product. The amplicons were verified on a 1% agarose gel and examined for non-specific banding.

The PCR amplicons were pre-treated with illustra^™^ ExoProStar 1-Step (GE Healthcare, Buckinghamshire, UK). Ten microlitres of the PCR product together with 2 μL of enzyme made up to a total volume of 14 μL with nuclease free water were incubated at 37°C for 20 min and inactivated for 20 min at 80°C. A total volume of 12 μL containing 10.8 μL of the cleaned amplicon and 1.2 μL of the Flav 100F forward primer (20 μM) were sent to the Australian Genome Research Facility (AGRF), Sydney, for Sanger sequencing using the ABI BigDye^®^ Terminator chemistry Version 3.1.

### Sequence analysis and phylogenetic tree construction

The partial sequences of the STRV isolates were compared against the NS5 gene region of the STRV prototype that was first isolated in 1961, GenBank reference STRV C338 KF917540 and KM225263. For completeness of study, comparisons were also made against the Kokobera virus (KOKV) AUS MRM32 (GenBank AY632541.4), Torres virus strain TS5273 (GenBank KC788513.1), a STRV-like virus; Bainyik virus strain MK7979 (GenBank KM225264.1) and New Mapoon virus strain CY1014 (KC788512.1). Data were subjected to the jModelTest 2.1.4 [33] to determine the appropriate mode of analysis. Phylogenetic analyses were conducted using Geneious 6.0.6 and *MEGA* version 5.10 [34]. The sequenced gene targets were aligned by the ClustalW Multiple Alignment Tool and edited to a final alignment of 556bp. A Bayesian Markov chain Monte Carlo (MCMC) analysis using MrBayes was used for phylogenetic inference [35]. The analysis was performed using a molecular clock probability distribution on branch lengths with gamma distributed rate variation among sites. MCMC analysis with four chains and temperature set to 0.2 were run for 1100000 cycles. The phylogeny construction of translated protein sequences were analysed by the ML method using the Jones-Taylor-Thornton (JTT) model for amino acid substitution [36]. Confidence limits were set on 1000 bootstrap replicates. A pairwise distance matrix was used to display distances between alignments. All STRV NS5 partial sequences in this study were deposited in GenBank (Accession no. KU059110—KU059152) (Table 1).

**Table 1:** Coordinates of mosquito collection sites on the NSW coast where Stratford virus was isolated, 1995–2013. Locations are listed in Table 2.

Collection Site	Coordinates [37]	
Byron Bay	28° 38' 29.080"S	153° 36' 43.026"E
Port Stephens	32° 42' 47.303"S	152° 03' 56.872"E
Parramatta	33° 47' 58.341"S	151° 00' 22.415"E
Lake Macquarie	33° 03' 42.281"S	151° 36' 05.239"E
Central Coast	33° 25' 30.728"S	151° 22' 46.844"E
Batemans Bay	35° 41' 25.072"S	150° 09' 34.965"E
Hawkesbury	33° 20' 25.760"S	150° 46' 58.488"E
West Pennant Hills	33° 38' 02.331"S	150° 58' 50.711"E
Penrith	33° 44' 59.987"S	150° 42' 01.544"E
Blacktown	33° 46' 00.002"S	150° 55' 00.935"E
Sydney Olympic Park	33° 50' 52.721"S	151° 04' 05.431"E
Georges River	33° 59' 15.243"S	151° 01' 30.671"E

## Results

A total of 43 isolates of STRV was detected in mosquitoes collected in coastal NSW between 1995 and 2013 (Table 1). The virus was isolated from mosquitoes collected in the far north coast (Byron Bay), mid-north coast and Hunter region (Port Stephens, Lake Macquarie, and Central Coast), Sydney metropolitan region (Blacktown, Georges River, Hawkesbury, Parramatta, Penrith, Sydney Olympic Park and West Pennant Hills) and south coast (Batemans Bay). No STRV was detected in mosquitoes collected at sampling sites west of the Great Dividing Range. There was a marked difference in the seasonal activity of STRV across the study period with sporadic detection of the virus from 1995 until 2006, when the first major season of STRV activity was recorded (n = 14), followed by notable peaks in 2010 (n = 11) and 2013 (n = 11) Table 2.

**Table 2:** The total number (n = 43) of Stratford virus isolates recorded by location and mosquito species from 1995 through 2013.

Isolate No.	Year Collected	Site of virus isolation	Mosquito species	GenBank Accession No.
23769	1995	Batemans Bay	*Aedes vigilax*	KU059132
23770	1995	Batemans Bay	*Aedes vigilax*	KU059133
33016	1996	Port Stephens	*Aedes vigilax*	KU059134
54892	1999	Parramatta	*Aedes notoscriptus*	KU059135
57653	1999	West Pennant Hills	*Aedes notoscriptus*	KU059136
92B0060	2002	Penrith	*Anopheles annulipes*	KU059152
72083	2005	Hawkesbury	*Aedes procax*	KU059137
76859	2006	Port Stephens	*Aedes procax*	KU059138
77538	2006	Port Stephens	*Aedes procax*	KU059139
78596	2006	Port Stephens	*Aedes procax*	KU059146
78618	2006	Port Stephens	*Aedes vigilax*	KU059147
78621	2006	Port Stephens	*Aedes vigilax*	KU059148
78563	2006	Port Stephens	*Aedes vigilax*	KU059145
78776	2006	Port Stephens	*Aedes vigilax*	KU059149
78202	2006	Lake Macquarie	*Aedes procax*	KU059142
77883	2006	Lake Macquarie	*Aedes procax*	KU059140
78224	2006	Lake Macquarie	*Aedes vigilax*	KU059143
78240	2006	Lake Macquarie	*Aedes vigilax*	KU059144
81272	2006	Lake Macquarie	*Aedes aculeatus*	KU059150
79463	2006	Lake Macquarie	*Aedes alternans*	KU059151
77970	2006	Port Stephens	*Aedes vigilax*	KU059141
160783	2010	Byron Bay	*Aedes notoscriptus*	KU059112
160375	2010	Byron Bay	*Aedes notoscriptus*	KU059110
162702	2010	Byron Bay	*Aedes notoscriptus*	KU059120
161325	2010	Port Stephens	*Aedes vigilax*	KU059114
161383	2010	Port Stephens	*Aedes vigilax*	KU059115
161114	2010	Port Stephens	*Aedes vigilax*	KU059113
162539	2010	Georges River	*Aedes vigilax*	KU059119
160641	2010	Georges River	*Aedes vigilax*	KU059111
161640	2010	Lake Macquarie	*Aedes vigilax*	KU059118
161572	2010	Batemans Bay	*Aedes vigilax*	KU059116
161575	2010	Batemans Bay	*Aedes vigilax*	KU059117
178964	2013	Central Coast	*Aedes procax*	KU059127
178003	2013	Central Coast	*Aedes vigilax*	KU059124
177701	2013	Blacktown	*Aedes procax*	KU059123
177238	2013	Blacktown	*Aedes procax*	KU059121
177296	2013	Blacktown	*Aedes procax*	KU059122
179388	2013	Penrith	*Aedes procax*	KU059131
178816	2013	Byron Bay	*Aedes procax*	KU059126
178803	2013	Byron Bay	*Aedes procax*	KU059125
179211	2013	Lake Macquarie	*Aedes procax*	KU059129
179071	2013	Lake Macquarie	*Aedes procax*	KU059128
179347	2013	Sydney Olympic Park	*Aedes procax*	KU059130

The majority of STRV isolates were from separate pools of *Ae*. *vigilax* (46.5%), *Aedes procax* (Skuse) (34.9%) and *Aedes notoscriptus* (Skuse) (11.6%), and these were across a range of geographic locations (Table 1). Three of the STRV isolates were also detected in *Aedes aculeatus* (Theobald), *Aedes alternans* (Westwood), and *Anopheles annulipes* Skuse. There was no discernible trend in the temporal association between mosquito species and STRV detection.

**Fig 1:**
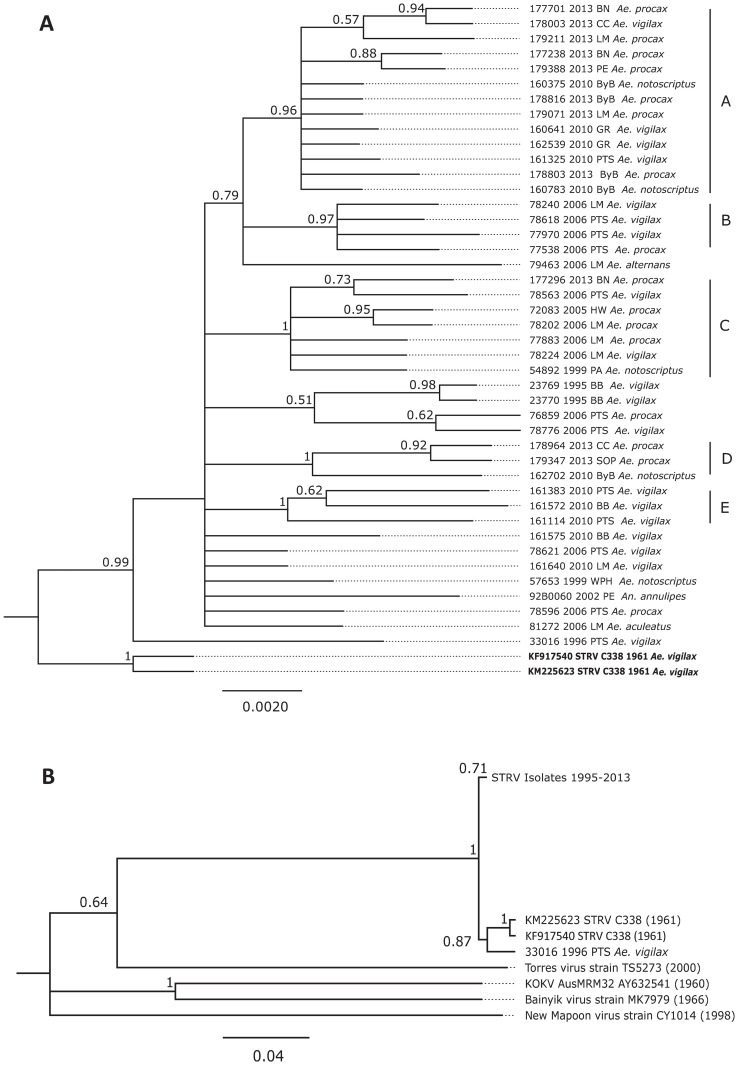
Phylogenetic association of STRV isolates (n = 43) and STRV C388 GenBank prototypes (A) and the association between STRV isolates, TS5273, KOKV AusMRM32, Banyik MK7979 and the New Mapoon CY1014 virus strain (B). The trees are based on the nucleic acid partial sequence of the NS5 gene region (556 bp). The posterior probability values are shown at the nodes with the branch lengths measured in the number of substitutions per site indicated by the scale bar. Abbreviations used: BN = Blacktown; CC = Central Coast; LM = Lake Macquarie; ByB = Byron Bay; PE = Penrith; PTS = Port Stephens; GR = Georges River; BB = Batemans Bay; SOP = Sydney Olympic Park; HW = Hawkesbury; PA = Parramatta; WPH = West Pennant Hills.

Examination of 43 sequenced isolates covered a total of 556 bp positions in the final data set. Temporal clustering between groups of some isolates collected from 2006, 2010 and 2013 are presented by clades A, B, C, D and E (Fig 1A) with posterior probability ≥ 0.96 for clustered nodes. Comparison between the STRV isolates and the 1961 STRV prototypes (GenBank KM225623; KF917540) showed a high level of support (0.99) with the nucleotide sequence of isolate 33016 showing the most number of nucleotide inconsistencies (n = 10–22). Excepting for Cluster B, no discernible clustering by geographic location or by mosquito species was evident.

The relationship between STRV isolates, STRV prototypes, Torres virus STRV-like strain TS5273, KOKV AusMRM32-AY632541.4 and other viruses in the KOKV group (Bainyik virus strain MK7979, KM225264.1 and New Mapoon virus strain CY1014, KC788512.1) is presented in Fig 1B. A sequence homology of 0.64 was evident between the STRV isolates, STRV C338 and the STRV-like TS5273.

**Fig 2:**
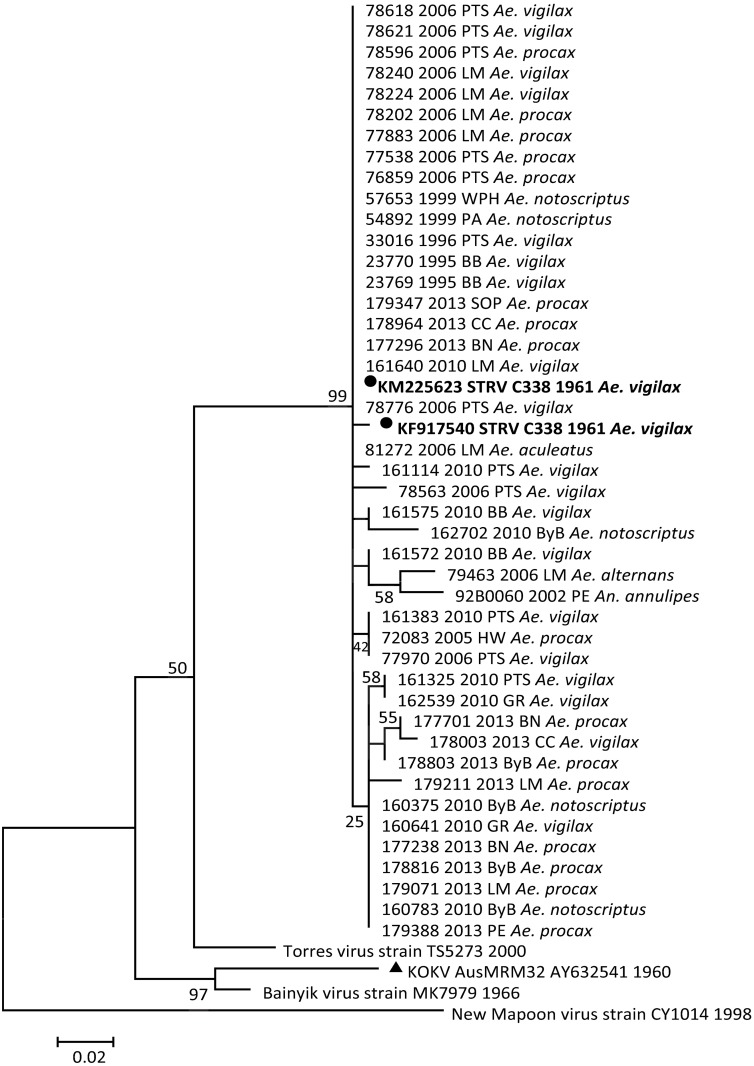
Phylogenetic association of deduced amino acids of the partial sequence of the NS5 gene region of STRV isolates (n = 43), STRV C338 ●, TS5273, KOKV AusMRM32 ▲, Bainyik MK7979 and New Mapoon CY1014 virus strain. The percentage association is shown above the branches with branch lengths measured in the number of substitutions per site indicated by the scale bar.

A pairwise comparison between test sequences and the STRV prototypes showed between 95.9% and 98.4% homology. The percentage identity between the STRV isolates, TS5273 and the KOKV AY632541.4 prototype was 76.4%– 78.3% and 74.4% -77.5%, respectively. Between 96.2%–100% genetic similarity was shown between STRV isolate sequences with up to 22 observed oligonucleotide differences. Comparison of the deduced amino acid sequences are shown in Fig 2. There were a total of 175 positions in the final dataset. No distinctive difference between STRV isolates was evident with 99% homology shown between STRV isolates and the STRV prototypes. However, 50% similarity was apparent between STRV isolates, the TS5273, KOKV AusMRM32 and the KOKV group of viruses. A paired comparison of amino acids between the STRV isolates alone was 95.8% with a total of eight variances shown, whereas 100% identity was evident between STRV isolates and STRV C388.

## Discussion

This study represents one of the first genetic analyses of STRV isolates distributed over a wide spatial and temporal scale in New South Wales. Surveillance of mosquito-borne pathogens has been underway across Australia for many decades but the isolation of STRV from field collected mosquitoes is not commonly reported. Isolations of STRV in this study were mainly from the *Aedes* species of mosquito. Similarly, Johansen and co-workers [38] detected a total of eight STRV isolates from *Aedes camptorhynchus* (Thomson) and *Aedes ratcliffei* (Marks) during arbovirus surveillance in 2002–2003 in Western Australia. In 2014, a surveillance program in South Australia detected only three isolates of STRV from one mosquito species, *Ae*. *notoscriptus* [39].

Sequence analysis of the 556 bp region encoding part of the methyltransferase and the start of the region encoding RNA-dependent-RNA polymerase in the NS5 gene sequence showed a sequence homology of 95.9%–98.4% between STRV isolates and STRV C338. In comparison, studies on isolates collected in Western Australia, and on isolates collected from NSW in 1995 (23759) and 1981(CS946) showed 97%–99% [38] and >97% [40] sequence homology, respectively.

Clustering of STRV isolates from 2010 and 2013 (Clusters A,D,E) was apparent, with collection sites spread over a wide area from the far north coast (Byron Bay), the mid-north coast and Hunter region (Lake Macquarie, Port Stephens and Central Coast) to the Sydney metropolitan area (Georges River and Blacktown). Other than Cluster B where three of the four isolates were collected within the same year (2006) from Port Stephens, there was no relationship between the geographic location and the temporal clustering of STRV sequences. This is unlike KOKV where three topotypes with both temporal and geographic clustering was established [41].

An overall divergence of 2% was shown between the STRV isolates and the C388 strain. This indicates a slow evolutionary change within the 43 STRV isolates collected over a period of 18 years in NSW from the first isolation of the virus in Cairns, Queensland, from *Ae*. *vigilax* in 1961[10]. However, the divergence shown between the STRV test isolates was higher at 4% with the greatest differences shown between the 1996 isolate and 2005, 2010 and 2013 isolates with distances of 13.00, 12.82 and 12.55, respectively. Isolate 33016, cultured from *Ae*. *vigilax* in 1996 from Port Stephens presented the least nucleotide similarity (96%). Consequently, eight of the STRV isolations from Port Stephens that were detected 10 years later (2006) showed sequence similarity in three isolates only (Cluster B). However, translation of the nucleic acids into proteins showed virtually no difference suggesting that there was no character change in phenotype.

The percentage identity shown between the STRV isolates, TS5273, and KOKV AusMRM32 was comparable to Nisbet et al. [31]. They found the nucleotide sequence of STRV to be closely related to TS5273 that was first isolated in 2000 from the Saibai Island and KOKVAusMRM32 with 74%–80% and 75% –76% nucleotide similarity, respectively. Subsequently, a species level cut-off of between 85% and 90% nucleotide identity has been proposed for species within the KOKV group [40]. Hence, this re-classifies STRV as a separate species. Overall, reports show little genetic change over a period of 34 years in the NS5/3' UTR region from the original C338 STRV isolated in Cairns in 1961 [40]. This similarity, often attributable to a common ancestor suggests that there is likely to be a single population of virus that may circulate within the study region, and across Australia, with annual activity driven by as yet undetermined environmental, entomological, or zoonotic factors. Moreover, although the variability between isolates was small in this study, genetic variability has the potential to drive activity. Furthermore, the NS5/3' UTR region is more conserved and therefore is likely to underestimate the variability in other regions.

The results of this investigation again identify mosquito species associated with coastal wetlands as playing a potentially important role in the transmission of mosquito-borne arboviruses [4]. The importance of STRV to public health has not been fully defined, yet serum antibody in Kokobera-like human infections have been recorded [11, 12], and the virus has been regularly isolated from mosquito species known to bite humans found in close association with urban habitats. The impact of STRV is not unlike Zika virus from its first identification in 1947 where initially, very little clinical importance was placed on the virus until the first recognized outbreak in 2007 from the island of Yap [42, 43], followed by the 2013–2014 and the 2015 outbreak in Brazil [44]. STRV was detected in only six mosquito species and little is known about its capacity to infect different mosquito species, or if it has any vector preferences. The ecology of the *Aedes* mosquitoes predisposes them to transmitting STRV to humans. The identification of reservoir hosts via blood meals has provided evidence that *Ae*. *notoscriptus* and *Ae*. *vigilax* occasionally feed on birds, although feeding on mammals is preferred [14, 45].

Notably, there were no isolates from *Culex annulirostris* Skuse during the study. This mosquito associated with freshwater habitats is considered the most important vector of endemic flaviviruses [4] and is generally thought to feed commonly on avian hosts [46]. The lack of STRV isolates from this mosquito species could be in part attributed to vector competence phenotype that is influenced by genetic factors and the environment [47]. Although the abundance of this mosquito is generally greater across inland regions, *Cx*. *annulirostris* was still common in coastal regions of NSW during the study period when favourable environmental conditions occurred, particularly during periods of above average rainfall and also in locations where STRV was isolated from other mosquito species [13].

Identifying host-seeking preferences of mosquitoes can potentially yield insights into important reservoir hosts of arboviruses. The mosquitoes from which STRV was isolated in this study have been identified as feeding on a wide range of mammal and avian hosts in urban environments [46, 48]. However, this investigation identifies the *Aedes* species of mosquitoes as being the main vector of Stratford virus with the first detection of the STRV prototype C338 in *Ae*. *vigilax* in 1961. Molecular studies have shown that there may be genetic differences between populations of *Ae*. *vigilax*, *Ae*.*notoscriptus* and *Cx*. *annulirostris* from different and potentially overlapping regions in Australia [49–52]. These genetic differences have been proposed as explanations of variable intra-specific vector competencies [50, 53] but may also influence other drivers in vector-pathogen-host transmission cycles such as host-seeking behaviour.

More research is required to determine if STRV exists in small cryptic foci across NSW or is introduced from elsewhere in the country by the movement of insects or vertebrate hosts. The genetic homology of STRV isolates suggests regular movement into coastal regions rather than isolated clusters of local activity. Notwithstanding the hosts, routes and potential environmental drivers of movement, drivers of local mosquito population abundance probably also play an important role in the activity of STRV. Population abundance of *Ae*. *vigilax* is dependent on the environmental drivers of temperature, rainfall and tides between seasons and regions across Australia [54–58] and is therefore difficult to predict. However, this mosquito is common during warmer months in many coastal regions with peak numbers differing between seasons. The environmental conditions for *Ae*. *procax* differ in that the availability of suitable habitats and seasonal distribution of rainfall primarily drives abundance. Consequently, numbers of this mosquito will vary between seasons, whereas the abundance of *Ae*. *notoscriptus* being primarily associated with urban habitats is generally consistent between seasons. Overall, while local environmental conditions may play a role in the activity of the identified vectors of STRV, there is currently no evidence that these conditions drive activity of the virus.

In the case of mosquito-borne viruses such as STRV that may not currently be associated with a significant public or veterinary health risk, understanding their genetic variability can provide insights into their vector and host associations, particularly as small genetic changes in the arboviruses can result in significantly enhanced virulence [59]. Future genetic studies should encompass a larger and less conserved region of the STRV virus genome such as the pre-membrane (prM) and Envelope (Env) genes that could be more informative in the study of evolutionary change and more meaningful in human infection. Viruses that are well adapted to a particular set of environmental conditions can perpetuate genetic qualities that reduce the genetic diversity within a population. Also, similarity between isolates can be attributed to constraints of the virus cycling between the mosquito vector and the natural host population [60–62]. In the absence of localized serological surveys of wildlife, it is difficult to identify the reservoir hosts of critical importance. The feeding habits of the *Aedes* species of mosquitoes suggests numerous host possibilities, from that of a mobile host, perhaps a bird or bat and mammals such as macropods may play a role in the dispersal of the virus into coastal regions of NSW.

Stratford virus activity was limited to the coastal regions of NSW and was primary vectored by the *Aedes* mosquito. The limited activity of STRV from inland regions of NSW and the absence of detection in the known flaviviruses vector *Cx*. *annulirostris* suggests that the virus may be generally limited in its distribution from coastal regions, differing to MVEV and WNV_KUN_ which are generally not associated with coastal regions [5, 63]. More research in tracking STRV is required to understand the evolution and host cycling of this endemic Australian arbovirus.
